# Supply kits for antenatal and childbirth care: a systematic review

**DOI:** 10.1186/s12978-017-0436-9

**Published:** 2017-12-13

**Authors:** Alicia Aleman, Giselle Tomasso, María Luisa Cafferata, Mercedes Colomar, Ana Pilar Betran

**Affiliations:** 1 Montevideo Clinical and Research Unit, Avda Italia s/n, Hospital de Clinical, 11600 Montevideo, Uruguay; 2 UNDP/UNFPA/UNICEF/WHO/World Bank Special Programme of Research, Development and Research Training in Human Reproduction, Department of Reproductive Health and Research, World Health Organization, Avenue Appia 20, 1211 Geneva, Switzerland

**Keywords:** Supply kits, Clean delivery kits, Pregnancy, Childbirth, Maternal mortality, Neonatal mortality

## Abstract

**Introduction:**

It is critical to increase the uptake of interventions proven to be effective to improve maternal and perinatal outcomes. Supply kits have been suggested to be a feasible strategy designed to ensure timely availability and effective follow-up of care.

**Objective:**

We conducted a systematic review to summarize the evidence on the uptake, effectiveness and safety of supply kits for maternal care.

**Search strategy:**

MEDLINE, the Cochrane Pregnancy and Childbirth Group’s Trials Register, Campbell Collaboration, Lilacs, Embase and unpublished studies were searched.

**Selection criteria:**

Studies that reported the efficacy, safety and use of supply kits for maternal healthcare were eligible. Participants were pregnant women or in childbirth. Supply kits were defined as a collection of medicines, supplies or instruments packaged together with the aim of conducting a healthcare task.

**Data collection and analysis:**

Two reviewers independently performed the screening, data extraction, and methodological and quality assessment.

**Main results:**

24 studies were included: 4 of them were systematic reviews and 20 primary studies. Eighteen studies evaluated a so-called “clean delivery kit”. In all but two studies, the kits were used by more than half of the participants. A meta-analysis was deemed inappropriate due to the heterogeneity in study design, in the components of the interventions implemented, in the content of the kits, and in outcomes. Nine studies assessed neonatal outcomes and found statistically significant reductions in cord infection, sepsis and tetanus-related mortality in the intervention group. Three studies showed evidence of reduced neonatal mortality (OR 0.52, 0.60 and 0.71) with statistically significant confidence intervals in all cases. Four studies reported odd ratios for maternal mortality, but only one showed evidence of a statistically significant decrease in this outcome but it was ascribed to hand washing prior to childbirth and not with the use of kits.

**Conclusion:**

This review suggests potential benefits in the use of supply kits to improve maternal and neonatal health. However, the observational nature of the studies, the heterogeneity and the use of kits incorporated within complex interventions limit the interpretation of the findings.

**Electronic supplementary material:**

The online version of this article (10.1186/s12978-017-0436-9) contains supplementary material, which is available to authorized users.

## Plain english summary

Supply kits are considered a potential strategy to improve maternal and child health, as they provide medication or diagnostic tests at the same time. This review searched for all published studies that tested the use of supply kits and assessed their effectiveness and safety. The studies found were of moderate to low quality. Most of them suggested benefits for mothers and babies, namely, reduced mortality and morbidity, with the use of supply kits. We concluded that although more research is needed to more comprehensively evaluate this strategy, it seems potentially useful for maternal and child health.

## Background

Improving maternal health and reducing child mortality were two of the eight Millennium Development Goals (MDGs) adopted by the international community in 2000. Between 1990 and 2015, the number of global maternal deaths dropped by 43% (from 532,000 to 303,000) [1]. To accelerate this decline, countries have established the Sustainable Development Goals (SDGs) as a follow-up initiative. Two targets are included under SDG 3: to reduce the global maternal mortality ratio to less than 70 per 100,000 births, with no country having a maternal mortality rate more than twice the global average, and to end preventable deaths of newborns, with all countries aiming to reduce neonatal mortality to 12 per 1000 live births [2].

Most maternal deaths are preventable, as the healthcare solutions to prevent or manage potential maternal complications are well known [1]. However, several factors can prevent the uptake of interventions that have been proven to be efficient and beneficial, specifically during pregnancy and delivery [3]. Supply chain deficiencies and stock-outs are among the most limiting barriers that hinder the delivery of effective practices in poor-resource settings [4–9]. Supply kits (packaged supplies targeting women, healthcare providers or health facilities) have been proposed to be a simple and low-cost intervention that can address various challenges routinely encountered in low-income countries. In the area of maternal and newborn health, supply kits have been designed to focus on issues ranging from timely availability of effective treatment in emergency situations and avoidance of stock-outs for routine care to achieving clean childbirth and reducing the incidence of infections and the associated complications, particularly in areas where women give birth at home [10–13]. Different types of supply kits have been implemented and tested as single or multicomponent interventions, and reviews assessing their effectiveness have been conducted, the latest of which was published in 2012 [14]. The timely update of the evidence is important for policy makers and implementers planning to use supply kits to improve care in areas where supply change deficiencies and stock-outs are a major bottleneck to reaching women with effective screening and treatment interventions. Even if the proportion of women delivering in health facilities is increasing, births often occur in a place with sub-standard hygienic conditions within the facility, without appropriately trained staff or without the appropriate medicines and conditions [15, 16].

The objective of this systematic review was to update and summarise the evidence with focus on the uptake, effectiveness and safety of supply kits for maternal care, particularly for antenatal and childbirth care at both institutional and community level.

## Methods

The methodology and reporting of results in this systematic review followed all steps proposed in the PRISMA statement [17]. This review was registered in the Prospero Centre for Reviews and Dissemination, University of York, with the number CRD42016043145 [18].

### Eligibility criteria of studies

#### Type of studies

Any study that reported the use of supply kits for maternal healthcare was eligible for inclusion, regardless of the study design, sample size, period and setting (e.g., nationwide, facility-based).

#### Type of participants

Pregnant women at any period of gestation or during labour and childbirth were eligible, regardless of women’s obstetric or medical characteristics, level of risk, education and socio-economic status.

#### Type of intervention

Supply kits. Kits were defined as a collection of medicines, supplies or instruments packaged together with the aim of conducting a healthcare task (e.g., antenatal care kit, caesarean section kit, delivery kit). This review included kits designed for individual use (e.g., kits given to each woman for childbirth at home) and kits designed for health facilities that contained supplies for use in their service population (e.g., all supplies necessary to conduct antenatal care for 100 women). Supply boxes of a single product (e.g., ARV) or kits that included only educational interventions were not included in this review.

#### Type of outcomes

The primary outcomes were as follows: maternal, perinatal and neonatal mortality; stillbirth (as defined by the authors); use of supply kits, including the proportion of women whose health care included application of the kits; number of ANC visits; low birth weight; complications of pregnancy, including prolonged anaemia, obstructed labour, eclampsia, postpartum haemorrhage, and postpartum depression (as defined by the authors); and referral to a health facility for any complication during pregnancy, delivery, or the postpartum period. The secondary outcomes included the following: iron/folate supplementation; tetanus toxoid immunization; institutional delivery; birth attended by a healthcare provider; use of bed nets (to avoid insect bites and prevent malaria); urine exams; syphilis and HIV diagnosis and treatment; initiation of breastfeeding within one hour of birth; wrapping babies within 30 min; and health care seeking for maternal and/or neonatal morbidities.

### Search strategy for identification of studies

The terms included in the search were medical supplies, clean, sanitary, disposable equipment, kit, birth kit, toolkit, package, box, prenatal care, antenatal care, pregnancy complications, pregnancy, postpartum period, labour, obstetric, intrapartum, partum, peripartum, and childbirth. An additional file shows the search terms in more detail [see Additional file 1]. Two experienced librarians in the Institute for Clinical Effectiveness (IECS, Buenos Aires, Argentina) and in the World Health Organization (WHO) assisted with the search. We searched MEDLINE (1966 to 2016), Embase (1980 to 2016) Lilacs (1982 to 2016), the Cochrane Pregnancy and Childbirth Group Trial Register and the Campbell Collaboration. Websites related to grey literature were also searched to identify unpublished studies. No limits regarding publication date or language were applied.

### Process of study identification, selection and data extraction

Citations identified through the search strategy of the electronic databases were imported into *Early Review Organizing Software* (EROS), and duplicates were deleted [19]. Four reviewers in pairs independently assessed the studies at each stage. In the first stage, all identified citations imported into EROS were screened based on the title and the abstract to select potentially relevant citations for full-text evaluation. When information in the title/abstract was insufficient to determine the inclusion/exclusion criteria, the full text was retrieved and evaluated. In the second stage, the full text of all the selected citations was retrieved and assessed. Citations fulfilling the inclusion criteria were included. Data were extracted using a structured data extraction form designed specifically for this review by the authors. Discrepancies were resolved through discussion and consensus. When data in the original publication were not sufficiently detailed, the authors were contacted for additional information.

### Risk of bias assessment

Both experimental and observational designs were eligible for inclusion in this review. We assessed the risk of bias of all included studies with quality assessment tools specifically intended for each study design using the currently internationally recommended tools. For experimental studies (randomized trials), we used the tool proposed by the Cochrane Collaboration to assess the quality of these types of studies [20]. The dimensions assessed with this tool are as follows: quality of randomization methods, allocation concealment, blinding of participants and of evaluators, incomplete outcome data and selective reporting of outcomes [20]. For observational studies, the assessment of the risk of bias considered three major criteria (methods for selecting participants, methods for measuring exposure and outcome variables, and methods to control for confounding) and two minor criteria (statistical methods and conflict of interest) [21–23]. For before and after studies, the assessment of the risk of bias considered blindness and reliability of the outcome measure and follow-up of participants [24, 25].

The quality of the systematic reviews was assessed using GRADE [26]. Primary studies included in the systematic reviews were evaluated for inclusion in our review, and those not retrieved in our search were included as appropriate.

### Analysis and reporting

The association between the use of supply kits and maternal, perinatal and neonatal outcomes was assessed using crude and adjusted odds ratios (ORs) and relative risks (RRs) with 95% confidence intervals or rates, depending on the study design. Meta-analyses were not performed due to the expected differences in the designs and data of the primary studies (different type of data, data collection, populations). The findings were reported considering two dimensions: the time when the supply kits were used (antenatal or during childbirth) and the outcomes related to the use of supply kits (uptake of the kits, maternal morbidity and mortality and neonatal morbidity and mortality).

## Findings

### Results of the search

**Fig. 1:**
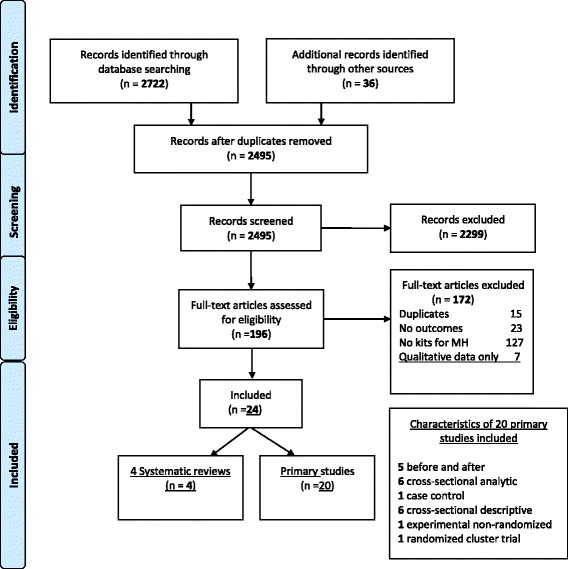
Flow Diagram

The search strategy identified 2495 unique citations. After assessing the titles and the abstracts for inclusion criteria, 2299 were excluded, and after a full-text evaluation, 172 additional citations were excluded. Finally, 24 manuscripts were selected, namely, 4 systematic reviews [14, 27–29] and 20 primary studies reporting data on the uptake, effectiveness and safety of the use of supply kits. All four systematic reviews included observational studies and searched for packaged interventions that included not only supply kits but also educational and behavioural components. The primary studies of the reviews were checked against our search results. One article [10] of the 20 primary studies included had not been identified through our search strategy and was thus added [See Additional file 2 for characteristic of the reviews]. Figure 1 shows the flowchart of our systematic review.

Table 1 and Table 2 show the characteristics of the 20 studies included in this review. The majority of the studies used a cross-sectional design (*n* = 12), and only one was a randomized controlled trial [30]. Most studies occurred in developing countries in Asia (*n* = 9) and Africa (*n* = 8). Two studies were from Oceania, and one study was conducted in the United Kingdom. The sample size of the studies ranged from 19 to 118,160 participants. In five studies, supply kits were the only component assessed by the authors, whereas 15 studies evaluated a multicomponent intervention that included the use of a clean delivery kit (CDK) together with an educational/behavioural component. All but one described kits designed for individual use either at home or in the health facility in the absence of complications. One study [11] used an emergency obstetric kit: a single-use code box that provided rapid access to effective treatment for specific emergencies.

**Table 1:** General characteristics of 20 Primary included studies

Characteristic	*N* (%)
Type of manuscript	
Articles in peer-reviewed journals	18 (90.0)
Congress Abstracts	2 (10.0)
Method	
Quantitative only	19 (95.0)
Both qualitative and quantitative	1 (5.0)
Study design	
Randomized controlled trial	1 (5.0)
Experimental non-randomized	1 (5.0)
Cross-sectional	12 (60.0)
Case Control	1 (5.0)
Before-and-after intervention	5 (25.0)
Region	
Asia	9 (45.0)
Africa	8 (40.0)
Europe	1 (5.0)
Oceania	2 (10.0)
Place of Delivery	
Health facility based	3 (15.0)
Home childbirth	7 (35.0)
Both (Home and Health facility)	10 (50.0)
Participants	
*Study subjects who received the intervention*	
Pregnant women	9 (45.0)
Birth attendants, health workers, TBAs	8(40.0)
Women during childbirth	5 (25.0)
Newborns	1 (5.0)
*Study subjects in which outcomes were assessed*	
Pregnant women	5 (25.0)
Women during delivery	17 (85.0)
Neonates	8 (40.0)
Women during postpartum	5 (25.0)
Infants	1(5.0)
Sample size	
> 10,000	5 (25.0)
10,000–1000	6 (30.0)
< 1000	9 (45.0)
Type of components	
Only the kits	5 (25.0)
Kits + behavioral intervention	4 (20.0)
Kits + behavioral + other components	1(5.0)
Kits + training	6 (25.9)
Kits + training + other components	4(20.0)

**Table 2:** Characteristics of 20 primary studies included

Author	Year	Number of participants	Country	Home or facility based	Type of study/design	Participants		Components of the Intervention (by study subjects)	Outcomes
						*Study subjects who received the intervention*	*Study subjects in whom the outcomes were measured*		
Balsara et al	2009	349 women	Egypt	Home 284 Health facility 65	Cross-sectional analytical	Health providers TBAs (Dayas) Pregnant women	Pregnant women Women during delivery	CDK (Health providers) CDK + training(TBAs) CDK+ use instructions (Pregnant women)	Number of ANC visits Use of CDK Clean delivery practices CDK acceptability
Calvert et al	2007	19 women	United Kingdom	Health facility	Cross-sectional study no analytic	Women in labor and delivery	Women in labor, delivery and postpartum	Homeopathic remedies kit (Women in labor and delivery)	Use of kit Benefit during birth
Darmstadt et al	2009	334 women 6 lost	Egypt	Home 276 Health facility 54	Cross-sectional study no analytic	TBAs (raedat, dayas, nurses) Community health workers Skilled birth attendants Pregnant women	Women during delivery Newborns	CDK (Health providers) CDK + training(TBAs) CDK+ instructions (Pregnant women)	Cord infection Puerperal infection
Dickerson et al	2010	980 women 378 outreach providers	Tibet	Home 452 Health facility 495	Cross-sectional study no analytic	Pregnant women Outreach Health workers Laypersons	Women during delivery	CDK + training (to all) + antenatal and postnatal micronutrient supplementation(women)	Use of CDK Use of beneficial practices Breast-feeding
Garner et al	1994	126 women 131 neonates	Papua New Guinea	Home	Before and after study	Pregnant women	Neonates	CDK+ use instructions (women)	Neonatal sepsis Use of the kits Fever
Greenwood	1990	15 villages with PHC and non-PHC; 673 women before the introduction of the Program and 1913 after	Gambia	Home	Before and after study	TBAs	Pregnant women Women during delivery Newborns Women during postpartum period	Training + obstetric package^a^(TBA)	Number of ANC visits Maternal mortality Maternal morbidity Perinatal mortality Stillbirth Neonatal death Tetanus immunization Pre-eclampsia
Hassan et al	2012	225 women 82 health workers	Pakistan	Home 100 Health facility 125	Cross-sectional, questionnaire study, no analytic^c^	TBA, women.	Women during delivery Health workers	CDK	Use of CDK Use of clean delivery practices
Jokhio et al	2005	19,557 women TBAs	Pakistan	Home	Experimental randomized cluster trial	TBAs in seven *talukas* (rural Larkana)	Pregnant women Women during delivery	CDK + training (TBAs) VS NO training/NO CDK	Perinatal mortality Maternal mortality hemorrhage obstructed labor puerperal sepsis eclampsia, abortion Referral
Kapoor et al	1991	7687 deliveries	India	Home 6652 Health facility 1035	Cross-sectional study no analytic	TBAs Pregnant women	Pregnant women Women during delivery Neonates	Training to conduct deliveries(TBAs) CDK + tetanus toxoid immunization + Training in Clean delivery practices (women)	Tetanus toxoid coverage Neonatal mortality Tetanus neonatal mortality
McDougal et al	2012	1545 women	Lesotho	Health facility	Before and after study	Pregnant women	Pregnant women Women during delivery Women during postpatum Infants	MPP^b^: pregnancy, Intrapartum and 7 day post-partum ARVs (women) + neonatal ARVs for 7 or 28 days(infants)	Number of ANC visits HIV Positive women with 4 antenatal visits HIV negative women with 4 ANC visits Children with DNA PCR test at 53 months
Meegan et al	2001	118,160 births	Kenya Tanzania	Home Health facility	Experimental non-randomized clinical trial	TBAs	Neonates	CDK + training (TBAs)	Neonatal tetanus Mortality under 6 weeks
Mukasa et al	2012	3116 women	Uganda Tanzania	Home Health facility	Cross-sectional study no analytic	Pregnant women	Women during delivery	CDK + misoprostol + information(women)	Use of CDK Acceptability
Ouma et al	2012	7080 deliveries before 8269 deliveries after	Kenya	Health facility	Before and after study	Birth attendants (BA)	Women with an obstetric emergency	Obstetric emergency kit (BA)	Maternal mortality due to hemorrhage, pre eclampsia/eclampsia, cardio pulmonary arrest
Quaiyum et al	2012	118,594 women	Bangladesh	Home	Before and after study	TBAs Pregnant women from selected *upazilas*	Women at delivery	CDK + misoprostol + blood collection mat + training (TBAs and women)	Maternal mortality
Raza et al	2013	420 neonates	Pakistan	Home 311 Health facility 109	Matched Case-control study	Cases: Newborns with tetanus Controls: Newborns without tetanus	No applicable	CDK	Use of CDK Use of hygienic practices
Seward et al	2015	40,602 deliveries	Nepal India Bangladesh	Home	Cross-sectional analytical	Women during delivery	Women during delivery	CDK training on clean delivery practices (hand washing)	Use of CDK Maternal mortality
Seward et al	2012	19,754 deliveries	Nepal India Bangladesh	Home	Cross-sectional analytical	Women during delivery	Neonates	CDK training on clean delivery practices	Neonatal mortality
Tsu	2000	1600 women	Nepal	Home	Cross-sectional analytical	Women who delivered a live newborn	Newborn	CDK	Cord infection Newborn infection Maternal infection
Vallely et al	2016	200 women	Papua New Guinea	Home 108 Health facility 92	Cross-sectional analytical	Pregnant women	Women during delivery Women during postpartum	CDK + training + misoprostol (women)	Use of CDK CDK acceptability
Winani et al	2007	3262 women	Tanzania	Home 1792 Health facility 1186	Cross-sectional analytical	Pregnant women	Women during delivery Women during postpartum Newborns	CDK + (women’s education)	Use of CDK Cord Infection Puerperal sepsis

^a^Obstetric package: clean dressing, scissors and string, oral ergometrine, disinfectant, color-coded spring balance for weighing newborns

^b^MPP: Minimum Prevention Mother to Child Transmission Package

^c^Mothers and health workers were asked details about their last delivery

In most of the studies, women, birth attendants, health workers or traditional birth attendants (TBAs) received a CDK intended for childbirth either at home or in the health facility (if the woman received the CDK and she delivered at a health facility, she brought the CDK with her to the health facility). Seven studies reported data on interventions that were implemented at home only. In 10 studies, the intervention was either at home or in health facilities, and in three, the intervention was only at the health facility. Nine studies reported neonatal and perinatal outcomes, eight reported maternal outcomes, and ten studies reported data on the uptake of the supply kits (See Table 1).

### Kits’ effectiveness and safety during pregnancy

Women were provided supply kits to be used during pregnancy in only one study (HIV-related kits) [12]. In all the other studies, the kits provided were intended for childbirth and were given either directly to the women or to the healthcare providers. They were provided either during pregnancy or at the time of birth. McDougal evaluated the effect of a kit of ARV drugs on preventing mother-to-child transmission of HIV in Lesotho (12). The kit, distributed at health centres for individual use, contained all the necessary pregnancy, delivery and early postnatal antiretroviral medications for the mother and the infant. No differences were found between the two groups of women before and after the intervention in terms of the coverage and quality of ANC or infant immunization within three months of birth (a proxy of HIV testing for babies). However, there was a significant reduction in HIV-positive women delivering in health facilities (57.7% pre-intervention and 48.9% post intervention, *p* < 0.05).

### Kits’ effectiveness and safety during childbirth and the immediate postpartum period

Nineteen studies evaluated the use of supply kits during childbirth and the immediate postpartum period. These were supply kits that aimed to promote clean delivery, and they focused predominantly on components to achieve the “6 cleans” proposed by the WHO (clean attendant hands, surface, blade, cord tie, towels to dry then wrap the baby, and cloth to wrap the mother) [31]. Table 3 displays the content of the supply kits in each study. All the supply kits were intended for vaginal delivery, with no studies evaluating supply kits for caesarean section. The supply kits were distributed at no cost in seven (35%) studies. Out-of-pocket payment was required in one study, while in another study, whether kits were charged for varied according to region. For the remaining 10 studies (50%), information on charges to the women was unclear.

**Table 3:** Components of the Kits

	Soap	Gloves	Clean plastic drape	Sterile razor	Cord tie/clamp	Gauze/cotton	Antiseptic	Newborn cap	Other	Cost of the kit
Pregnancy										
Mc Dougal 2012									HIV treatment	unknown
Childbirth										
*Clean Delivery Kits*										
Balsara 2009	x		x	x	x					free
Calvert 2007									homeopathic remedies	unclear
Dickerson 2010	x	x	x	x	x	x		x	vitamins-micronutrients	probably
Darmstadt 2009		x	x	x	x	x	x			free
Garner 1994				x	x		x			free
Hassan 2012	x	x	x	x	x	x	x	x		unclear
Meegan 2001				x	x		x			free
Jokhio 2005	x	x		x	x	x	x			free
Kapoor 1991		x	x	x						unclear
Mukasa 2012	x	x	x	x	x	x			misoprostol-blood collection mat	unclear
Quaiyum 2012^a^									misoprostol	unclear
Raza 2013	x	x	x	x	x					unclear
Seward 2012	x		x	x	x	x				free/very low cost
Seward 2015	x		x	x	x	x				free/low cost
Tsu 2000	x		x	x	x					free or cost depending on region
Vallely 2016	x	x	x	x	x				misoprostol	free
Winani 2007	x		x	x	x					free
*Emergency kits*										
Ouma 2012										not applicable
*Obstetric care kit*										
Greenwood 1990									Scissors, string, dresses, oral ergometrine, balance	unclear

^a^Components of the clean delivery kit are not described

Ten studies including 53,068 women reported data on the uptake of the use of supply kits [30, 32–40] (See Table 4). The median use of supply kits was 62%, ranging from 15% to 100%. Studies that reported the uptake of supply kits both at home and at a health facility found that the use of kits for births at home was always higher than the use at health facilities [4, 32]. One study reported a high acceptability and use but did not share the data [41]. Uptake or impact of the supply kit according to the receiver (e.g., health provider or women) was not addressed specifically in any study.

**Table 4:** Frequency of use of clean delivery kits

	Global n/N (%)	Home n/N (%)	Health facility n/N (%)
Balsara 2009	248/349 (71.0)	214/284 (75.4)	44/65(67.7)
Calvert 2007			
Before labour	12/19 (63.1)		
During labour	15/19 (78.9)		
Pospartum	19/19 (100.0)		
Dickerson 2010	932/962 (96.9)		
Garner 1994			
Razor pack	22/33 (66.7)		
Clamp pack	17/34 (50.0)		
Hassan 2012	72/225 (32.0)^a^		
Jokhio 2005	8172/10114 (80.0)		
Raza 2013			
Cases	24/123 (17.1)		
Controls	99/280 (35.4)		
Seward 2015	5210/34660 (15.05)		
Vallely 2016	115/200 (57.5)	99/106 (93.4)	16/94 (17.0)
Winani 2007	1820/3058 (59.5)		

^a^Based on women’s report

The studies included in this review reported the effect of the intervention as a whole, regardless of the number and type of components (Table 1). We could not separate the effect of the different components, and thus the effect estimates reported hereafter refer to the complete intervention, not exclusively to the supply kits. Only five studies assessed the effect of the supply kits alone. One evaluated an emergency kit [11], one a homeopathic delivery kit [33], and three assessed the effect of CDK [35, 37, 42]. The latter three evaluated different outcomes, but in all cases, they reported an increase in the use of clean delivery practices with the use of the kits.

Nine studies reported data on the effect of CDK on neonatal outcomes [10, 13, 30, 35, 40, 42–45] (See Table 5). The only randomized controlled trial in the review reported statistically significant reductions in perinatal and neonatal mortality in the arm that used kits in the context of a complex intervention. Of the other eight observational studies, three showed a protective effect regarding cord infection in the kit group compared to the control group. A protective effect was also reported for tetanus-specific mortality, neonatal sepsis and neonatal mortality. However, in addition to the observational nature of the study, it tested a complex intervention, and thus it was not possible to attribute the reductions in mortality to the use of the supply kit [30].

**Table 5:** Neonatal outcomes

	Intervention group	Control group	OR adjusted IC 95%
Cord infection			
Darmstadt 2009	14/235	13/93	0.42 (0.18–0.97)
Tsu 2000	–	–	0.45 (0.25–0.81)
Winani 2007^a^	3/1820	48/1238	0.04 (0.01–0.12)
Sepsis			
Garner 1994^a^	1/67	8/64	0.11 (0.01–0.87)
Seward 2012	–	–	0.28 (0.12–0.65)
Tetanus-specific mortality^b^			
Kapoor 1991	0	14.6	–
Meegan 2001^c^	0.75	82	–
All-cause neonatal mortality			
Kapoor 1991^b^	19.9	39.2	–
Seward 2012	–	–	0.51(0.35–0.76)
Jokhio 2005	37	53	0.71 (0.62–0.83)
Greenwood 1990^a^	54/1159	47/675	0.65 (0.44–0.98)
Stillbirth			
Greenwood^a^	61/1220	37/712	0.96 (0.63–1.46)
Perinatal mortality			
Jokhio 2005^b^	85	120	0.70 (0.59–0.82)
Greenwood 1990^a^	99/1220	63/712	0.91(0.65–1.27)

^a^Unadjusted

^b^per 1000 livebirths

^c^before and after study

Eight studies reported data on the effect of kits on maternal outcomes [10, 11, 13, 30, 38–40, 46] (See Table 6). They were all community-based interventions except for the study by Ouma et al. [11], which was a study on an emergency kit for use in facilities. Three studies [13, 30, 40] reported lower puerperal infection in the intervention group, although only two were statistically significant, including the randomized controlled trial (which tested a complex intervention and thus multiple components, not only kits) [30]. Postpartum haemorrhage was also lower in the intervention group in three studies [11, 30, 39]. Maternal mortality was lower in the intervention group in three of the five studies that measured this outcome [11, 30, 46], but the difference was not statistically significant in any of them. One of these five studies [38] also tested the effect of hand washing separately from the supply kits. Hand washing prior to delivery independently reduced maternal mortality (OR 0.51 IC 95% 0.28–0.93).

**Table 6:** Maternal outcomes

	Kits group	Control group	OR adjusted IC 95%
Puerperal infection			
Jokhio 2005	78/10093	400/9432	0.18 (0.14–0.22)
Darmstadt 2009	1/235	4/93	0.11 (0.01–1.06)
Winani 2007	19/1798	50/1380	0.28 (0.17–0.48)
Post partum hemorrhage			
Vallely 2016	15/112	33/88	0.25 (0.13–0.52)^a^
Jokhio 2005+	174/10093	259/9432	0.62(0.51–0.75)^a^
Ouma 2012^b^	6/19 (31.6%)	14/27 (51.9%)	
Eclampsia			
Jokhio 2005^d^	23/10093	29/9432	0.74 (0.42–1.28)^a^
Maternal mortality			
Seward 2015	–	–	1.26 (0.62–2.56) / 0.51 (0.28–0.93)^e^
Ouma 2012	19/8120	27/6935	0.60 (0.33–1.08)
Quaiyum 2012^c^	137	338	–
Jokhio 2005	–	–	0.74 (0.45–1.23)
Greenwood 1990	13/1236	7/727	1.09(0.94–2.93)^a^

^a^Unadjusted OR

^b^The denominators are maternal deaths

^c^per 100,000 livebirths

^d^These outcomes were more related to the training component than the CDK use

^e^Use of kits with all components did not show difference with no kits use. The effect of hand washing prior delivery did show a significant reduction in the odd of maternal death

Ouma et al. implemented an obstetric emergency kit (called E-kit) for the treatment of postpartum haemorrhage, pre-eclampsia/eclampsia and cardiopulmonary arrest in health facilities using a before-and-after design [11]. They reported 27 maternal deaths in the year preceding the introduction of the E-kits and 19 in the first year of the E-kit implementation (among users and nonusers of kits). In the second year of the E-kit implementation, deaths from haemorrhage decreased by 31.6% overall and there were no maternal deaths in women treated with kits.

Regarding safety, none of the studies reported outcomes that could represent a harm to women or neonates in relation to the use of kits.

### Risk of bias

The quality assessment of each included study is presented in Additional file 3. A summary of the methodological quality assessment of risk of bias is presented for each domain (conflict of interest, control of confounders, methods and outcomes measure, and selection of participants) and design [See Additional file 4].

Two experimental studies were included in the review [30, 45], although only one was randomized [30]. Randomization, allocation concealment, blinding of assessment, incomplete outcome data and selective reporting showed low risk of bias. There was no blinding of participants in 80% of the quasi-experimental studies [10–12, 35, 45], and 80% had low risk of bias regarding the evaluation and measure of the primary outcome. Regarding the follow-up of participants and providers, there was a low risk of bias in 50% and 75% of the included studies, respectively. Observational studies with and without a comparison group [13, 32–34, 36, 38–44, 46] had a low risk of bias regarding the selection of participants (82%) and conflict of interest of authors (91%). The methods and measurement of the outcomes (information and detection bias) had a low risk of bias in 55% of studies as well as control of confounders. In summary, almost all studies included in this review were observational, and half of them had moderate to high risk of bias in three main dimensions: methods, measurement of outcomes and adjustment for confounders. Thus, the body of evidence collected was weak in terms of quality.

## Discussion

This systematic review identified 24 manuscripts, of which four were systematic reviews and 20 primary studies (primary data collection or secondary data analyses) presenting information on the uptake and impact of supply kits during pregnancy, childbirth or immediate postpartum period. The majority involved single-use kits for clean childbirth and infection prevention. Most studies were published in peer-reviewed journals, had a sample size of less than 1000 women and were observational studies. Only one study was a randomized controlled trial.

In most of the studies, the kits were used by more than half of the participants, with higher use shown at home than at the facility. In general, the group using the supply kits showed better measured outcomes. However, most of the included studies assessed the use of kits in the context of a complex intervention, and thus it was impossible to conclude that the kits were responsible for the observed differences. The groups using supply kits showed a statistically significant positive effect on neonatal outcomes; including reduced cord infection [13, 40, 42], sepsis [35, 43], neonatal mortality [10, 30, 43] and perinatal mortality [30]. Several studies showed a statistically significant decrease in maternal adverse outcomes in the group using supply kits (see Table 6) including reduction in puerperal infection [13, 30, 40] and postpartum haemorrhage [11, 30, 39] and a non-significant reduction in maternal mortality [11, 30, 46].Meta-analysis was deemed inappropriate due to the heterogeneity in the study designs, in the content of the supply kits, in the definition of compliance in use of supply kits and in the outcomes measured.

One study depicted potential adverse effects related to supply kits: a decrease in institutional childbirth in HIV-positive women who received the kit containing antiretroviral medication. Nonetheless, HIV-positive women compose a very specific group of women and might have different behaviours related to seeking antenatal and delivery care compared to the others [12].

This review showed that the most tested type of supply kit was the CDK. Eighteen studies evaluated kits with all or some of the components of the CDK (Table 2) and showed positive results in increasing clean delivery practices (Table 4). It has been estimated that clean childbirth practices could avert 6–9% of the 1.16 million newborn deaths in sub-Saharan African countries [31]. However, controversy remains regarding the independent effect of each component or clean practice. Seward et al. [38] conducted the only study that separately examined the effect of kit use and hand washing. They found that while hand washing with soap before delivery was independently associated to a reduction in maternal mortality, kit use had no significant effect on the prevention of maternal deaths. Similarly, Tsu et al. [42] reported that hand washing with soap before cutting the cord vs. not washing hands reduced cord infection even more than using a complete CDK. Balsara et al. [32] found no difference between kits’ users and non-users with regard to whether the birth occurred on a clean or unclean surface. These findings suggest that not all CDK components are used or have the same value in improving outcomes. Cultural beliefs may play a role in the understanding of the benefits of using certain components. For example, it could be difficult to understand the usefulness of laying down a plastic sheet during childbirth for women accustomed to delivering in a vertical position [41]. In addition, in our review, only five studies evaluated the effect of supply kits alone, and these studies were all heterogeneous (See Table 1).

Compliance with the “use of CDK” is not defined in the same way across studies, and this adds uncertainty to the conclusions. For example, Winani et al. [40] considered three different scenarios to define when a woman had “used a kit”: 1) if she used the full CDK or at least the plastic sheet and either the razor blade or cord tie (or both); 2) if she used the razor blade and cord tie only; or 3) if she used the razor blade alone. This resulted in heterogeneity and weakened the conclusions. In addition, the content of supply kits somewhat varied (see Table 3). However, considering the objective and concept of the kits, the components most frequently included were the sterile razor (*n* = 15 kits), cord tie (*n* = 14 kits) and soap (*n* = 11 kits).

In this review, all studies except one were conducted in developing countries, mainly in Africa and Asia. In low-resource settings, the use of supply kits is intended to facilitate the provision of interventions by providing all the resources needed for a given situation at one time. In addition, it optimizes the scarce contact between women and the healthcare system in areas where barriers related to accessibility, knowledge and lack of satisfaction prevent women from engaging with the system. In countries with a high prevalence of home births, CDK may be an effective option for reducing newborn infections as well as puerperal sepsis or other genital tract infections following childbirth. The successful implementation of supply kits minimizes the burden of having to separately acquire its contents, which would require knowledge of what is needed and how to use it. This is likely an unrealistic expectation in these settings. The challenges mentioned above justified the implementation of these studies in settings where maternal and neonatal mortality remains high.

We found only one study that described the use of a kit designed to address stock-outs and deficiencies in procurement at the facility level [11]. This kit aimed to address the delay in accessing treatment for obstetric emergencies at the health facility level once women were in the facility by considering the context of a dysfunctional supply chain and busy maternity units. In countries with weak and overstretched health systems, stock-outs in medicines and equipment occur routinely and hinder the delivery of effective practices even when women reach the health facility. These kits, which are not intended for individual use, may contain all the supplies required to attend 100 births or to conduct 100 ANC visits, for example. This type of kit has been tested recently in Mozambique in a WHO cluster randomized controlled trial [47]. Further research on the effectiveness and feasibility of this type of kit is warranted because procurement inefficiencies are a chronic and contemporary challenge in many low-resource settings and remote areas.

Even when the acceptability is high and when kits include instructions, the distribution of kits do not guarantee its use. Different studies have employed several approaches to provide access to supply kits including distribution through health facilities, community health workers, TBAs and private providers, such as pharmacists. However, the provider-specific impact on the uptake of supply kits has not been studied. Seward et al. [38] discussed the effect of a complex intervention using a participatory learning and action cycle with women’s groups in India and Nepal [38, 48, 49]. The discussions of clean delivery and care-seeking behaviours in women’s groups showed an increased uptake in reporting the use of kits in the intervention arm, compared to the control arm.

Available qualitative data is aligned with our results [50]. The main barriers to the implementation of supply kits are those related to socio-cultural and popular beliefs that birth preparation could bring bad luck. Financial constraints and a limited understanding of the instructions on how to use the supply kits have been also identified as accessibility barriers. On the other hand, convenience, the perception of the components as hygienic, and avoidance of delays in receiving care were viewed as satisfactory features that would incentivize the use of the supply kits.

Although four reviews have been previously published on this topic, this systematic review includes an additional four years of evidence, which combined with the current available qualitative evidence [50], it adds a more comprehensive approach to pre-existing knowledge. When the evidence on effectiveness is substandard and based on observational studies, the use of other types and sources of information can be critical to gain a better understanding of the intervention and the pathways of action. This is even more important for complex interventions. In addition, previous reviews have focused on the use of supply kits to reduce neonatal mortality and morbidity [28, 29], kits to conduct family and community interventions [27] and kits designed exclusively for birth [14]. Our review included supply kits for pregnancy and a larger range of maternal and neonatal outcomes.

### Strength and limitations

This review has several strengths. We developed a broad search strategy that included manuscripts and documents not published in peer-reviewed journals. We included studies with a variety of methodological approaches. As the studies originated from different settings in developing countries, we believe that the results of this review can be generalized to low-income settings. This review, however, has some limitations. Importantly, the studies retrieved were mostly observational or had weak experimental designs, which provide a low level of evidence compared to randomized controlled trials. Moreover, the supply kits in these studies were implemented in the context of complex interventions of varying degrees, and it was thus impossible to identify the “active ingredient/s” of the overall intervention. The outcomes were measured in different ways and units, making it difficult to arrive to reliable conclusions. The heterogeneity in the design of the supply kits and its components as well as the definition of compliance of the “use of kit” may hinder the comparability. Lastly, data collection was not always of high quality, and biases may have been present. Most of the studies included in this review were conducted in Africa and Asia, and their results may not be generalizable to other regions such as Latin America.

## Conclusions

Studies found in this review reported a reduction in maternal and neonatal morbidity (particularly infection-related morbidity) and neonatal mortality in the groups using supply kits. However, these findings should be interpreted with caution because virtually all the evidence was derived from observational studies and was thus prone to bias and because the effects observed cannot be ascribed to the supply kits alone, given their inclusion in multi-component interventions. The potential bias of the studies analysed and the heterogeneity hinder the reliability of identifying an overall effect of the kits. It would appear appropriate to continue its use and to expand to other preventive or therapeutic interventions with the inclusion of strong monitoring and evaluation strategies that include comparisons to control groups, as these methods could provide more evidence regarding the real effect of the kits.

### Implications for research

Sub-standard pre-conceptional and ANC care makes it difficult to diagnose and treat conditions occurring before or during pregnancy that can affect newborns or place women at increased risk of severe morbidity or mortality. These conditions include syphilis, hepatitis B, HIV, Group B streptococcus, malaria, and even hypertensive disorders of pregnancy, anaemia and urinary infections. A diagnostic and therapeutic package could increase accessibility and compliance. However, these types of kits have not been fully assessed, and research is needed to evaluate their potential effectiveness.

We found one study that implemented emergency kits. Life-threatening conditions during pregnancy and delivery could be effectively diagnosed and treated with specifically designed kits that ensure immediate emergency care. Further research is warranted in this area. Similarly, supply kits for caesarean sections have not been assessed. Finally, although supply kits designed for birth seem to be an effective strategy to improve maternal and neonatal health, there are still several questions that need answering. How and when supply kits should be distributed, who should receive them and what types of promotion and training strategies should be developed for their uptake remain unknown.

### Implications for practice

Positive effects were reported in many of the included studies. Although the evidence comes from observational studies, the use of supply kits could be an appealing feasible strategy for facilitating clean birth practices and access to certain commodities in low-resource settings. Implementation of this strategy requires low-complexity resources and could have a large impact, making supply kits an attractive alternative to increase the quality of care during pregnancy, delivery and the neonatal period, particularly at the community level in low-income countries. Nevertheless, even though the desirable effects seemed to outweigh the adverse effects, close surveillance of these kits should be considered because based on the low quality of evidence found in this review of studies, the authors cannot conclude without a doubt that supply kits are effective in reducing morbidity and mortality.

## Additional files

Additional file 1: Annex I. Search terms for search strategy. (DOCX 15 kb)

Additional file 2: Annex II. Primary studies included in Systematic Reviews retrieved by search strategy. (DOCX 14 kb)

Additional file 3: Annex III- Quality assessment by individual study. (DOCX 54 kb)

Additional file 4: Annex IV. A summary of methodological quality assessment of risk of bias by study design. (DOCX 192 kb)
