# Supplementary material for: Supply kits for antenatal and childbirth care: a systematic review
Source: Reprod Health. 2017 Dec 13;14:175. doi: 10.1186/s12978-017-0436-9 (PMC5729253; doi:10.1186/s12978-017-0436-9)
Supplement: Supplementary file 2 — Annex II. Primary studies included in Systematic Reviews retrieved by search strategy. (DOCX 14 kb) [file 12978_2017_436_MOESM2_ESM.docx]

**Annex II. Primary studies included in Systematic Reviews retrieved by search strategy**

| **Author** | **Inclusion criteria** | **Included studies** | **Studies previously included in our review** | **New included studies** | **Studies not included in our review** | **Reason for exclusion** |
| --- | --- | --- | --- | --- | --- | --- |
| Hundley 2012 | Intervention: birth kit defined as a kit intended for use in the intrapartum period, specifically at the actual delivery or birth of the baby. | Kapoor 1991 Garner 1994 Tsu 2000 Meegan 2001 Jokhio 2005 Path 2005 Mullany 2006 Tielsch 2007 Winani 2007 Balsara 2009 Darmstadt 2009 | Kapoor 1991 Garner 1994 Tsu 2000 Meegan 2001 Jokhio 2005 Path 2005 Tielsch 2007 Winani 2007 Balsara 2009 Darmstadt 2009 | None | Mullany 2006 | Kits are provided to both groups (intervention and control) |
| Haws 2007 | Only studies from developing countries testing packages of health interventions (i.e. more than one component intervention) were considered eligible for inclusion. | Kappor 1991 Jokhio 2005 Greenwood 1990 Meegan 2001 | Kappor 1991 Jokhio 2005 Meegan 2001 | Greenwood 1990 |  |  |
| Bhutta 2005 | Intervention: Care packages | Meegan 2001 Tsu 2000 Kapoor 1991 Bang 1999 Garner 1994 | Meegan 2001 Tsu 2000 Kapoor 1991 Bang 1999 Garner 1994 | None | None |  |
| Schiffman 2010 | Intervention: Community based intervention packages | Bang 2005 Kumar 2008 Darmstad 2010 Baqui 2008 Carlo 2010 Jokhio 2005 Manandhar 2004 | Jokhio 2005 | None | Bang 2005 Kumar 2008 Darmstad 2010 Baqui 2008 Carlo 2010 Manandhar 2004 | Kumar 2008 and Baqui 2008 do not evaluate kits  Carlo 2010 and Bang 2005 evaluate neonatal kits, Darmstad 2010 and Manandhar 2004 promote several beneficial practices and use of kits are one of them but are not evaluated individually. |
