# Supplementary material for: Supply kits for antenatal and childbirth care: a systematic review
Source: Reprod Health. 2017 Dec 13;14:175. doi: 10.1186/s12978-017-0436-9 (PMC5729253; doi:10.1186/s12978-017-0436-9)
Supplement: Supplementary file 3 — Annex III- Quality assessment by individual study. (DOCX 54 kb) [file 12978_2017_436_MOESM3_ESM.docx]

**Annex III- Quality assessment by individual study**


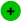
 : low risk of bias


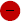
 : high risk of bias

: moderate risk of bias


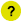
 : unclear risk of bias

**Case-control studies (1)**

| **ID** | **Selection of participants (selection bias)** | **Comparability between groups (selection bias)** | **Control of confounders** | **Statistical methods (excluding control of confounders)** | **Methods and outcome measures (detection and reporting bias)** | **Conflict of interest** |
| --- | --- | --- | --- | --- | --- | --- |
| Raza 2013 | 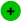 | 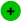 | 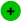 | 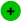 | 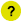 | 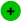 |

**Before and after studies (5)**

| **ID** | **Blinded assessment of the primary outcome (detection bias)** | **Reliable of the primary outcome measures** | **Follow-up of the providers (attrition bias)** | **Follow- up of the participants** |
| --- | --- | --- | --- | --- |
| Ouma 2012 | 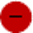 | 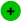 | n/a | 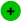 |
| Quaiyum 2012 | 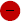 | 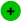 | n/a | 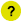 |
| Garner 1994 | 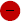 | 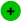 | n/a | 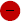 |
| McDougal 2012 | 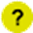 | 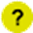 | n/a | 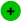 |
| Greenwood 1990 | 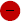 | 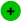 | n/a | 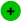 |

**Cross-sectional (analytical) (6)**

| **ID** | **Selection of participants (selection bias)** | **Methods and outcome measures (detection and reporting bias)** | **Control of confounders** | **Conflict of interest** |
| --- | --- | --- | --- | --- |
| Balsara 2009 | 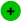 | 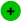 | 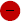 |  |
| Seward 2015 | 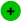 | 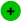 | 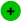 | 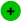 |
| Seward 2012 | 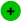 | 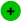 | 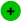 | 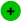 |
| Tsu 2000 | 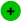 | 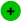 | 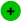 | 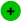 |
| Vallely 2016 | 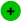 |  | 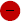 | 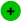 |
| Winani 2007 | 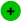 | 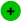 | 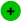 | 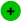 |

**Descriptive Cross sectional (6)**

| **ID** | **Selection of participants (selection bias)** | **Methods and outcome measures (detection and reporting bias)** | **Control of confounders** | **Conflict of interest** |
| --- | --- | --- | --- | --- |
| Calvert 2007 |  |  | 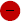 | 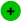 |
| Darmstadt 2009 | 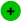 |  | 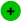 | 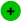 |
| Dickerson 2010 | 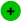 | 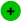 | 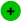 | 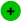 |
| Hassan 2012 |  |  | 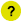 | 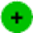 |
| Kapoor 1991 | 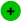 | 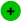 | 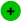 | 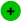 |
| Mukasa 2012 | 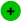 | 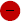 | 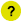 | 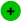 |

**Randomized cluster trial (1)**

| **ID** | **Random sequence generation (selection bias)** | **Allocation concealment (selection bias)** | **Blinding of participants and personnel (performance bias)** | **Blinding of outcome assessment (detection bias)** | **Incomplete outcome data (attrition bias)** | **Selective reporting (report bias)** | **Other bias** | **Conflict of interest** |
| --- | --- | --- | --- | --- | --- | --- | --- | --- |
| Jokhio 2005 | 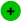 | 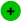 | 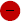 | 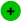 | 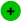 | 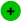 | 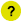 | 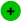 |

**Non randomized Controlled trial (1)**

| **ID** | **Random sequence generation (selection bias)** | **Allocation concealment (selection bias)** | **Blinding of participants and personnel (performance bias)** | **Blinding of outcome assessment (detection bias)** | **Incomplete outcome data (attrition bias)** | **Selective reporting (reporting bias)** | **Other bias** | **Conflict of interest** |
| --- | --- | --- | --- | --- | --- | --- | --- | --- |
| Meegan 2001 |  |  | 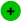 | 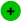 | 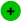 | 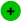 | 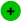 | 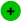 |

Mixed **study (1)**

| **ID** | **Clarity of the research question** | **Context** | **Appropriate design to answer the research question** | **Bias assessment** | **Sampling** | **Data collection and analysis** | **Relevance** |
| --- | --- | --- | --- | --- | --- | --- | --- |
| McDougal 2012 | 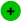 | 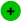 | 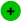 | 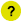 | N/A | 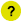 | 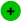 |
