# Supplementary material for: Supply kits for antenatal and childbirth care: a systematic review
Source: Reprod Health. 2017 Dec 13;14:175. doi: 10.1186/s12978-017-0436-9 (PMC5729253; doi:10.1186/s12978-017-0436-9)
Supplement: Supplementary file 4 — Annex IV. A summary of methodological quality assessment of risk of bias by study design. (DOCX 192 kb) [file 12978_2017_436_MOESM4_ESM.docx]

Annex IV. A summary of methodological quality assessment of risk of bias by study design
